# Using evolutionary demography to link life history theory, quantitative genetics and population ecology

**DOI:** 10.1111/j.1365-2656.2010.01734.x

**Published:** 2010-11

**Authors:** Tim Coulson, Shripad Tuljapurkar, Dylan Z Childs

**Affiliations:** 1 Department of Life Sciences, Imperial College LondonSilwood Park SL5 7PY, UK; 2 Department of Biology, Stanford UniversityStanford, CA 94305-5020, USA; 3 Department of Animal and Plant Sciences, The University of SheffieldSheffield S10 2TN, UK

**Keywords:** age-stage structure, integral projection models, ontogenetic development, reproductive allocation, Soay sheep

## Abstract

**1.** There is a growing number of empirical reports of environmental change simultaneously influencing population dynamics, life history and quantitative characters. We do not have a well-developed understanding of links between the dynamics of these quantities.

**2.** Insight into the joint dynamics of populations, quantitative characters and life history can be gained by deriving a model that allows the calculation of fundamental quantities that underpin population ecology, evolutionary biology and life history. The parameterization and analysis of such a model for a specific system can be used to predict how a population will respond to environmental change.

**3.** Age-stage-structured models can be constructed from character-demography associations that describe age-specific relationships between the character and: (i) survival; (ii) fertility; (iii) ontogenetic development of the character among survivors; and (iv) the distribution of reproductive allocation.

**4.** These models can be used to calculate a wide range of useful biological quantities including population growth and structure; terms in the Price equation including selection differentials; estimates of biometric heritabilities; and life history descriptors including generation time. We showcase the method through parameterization of a model using data from a well-studied population of Soay sheep *Ovis aries*.

**5.** Perturbation analysis is used to investigate how the quantities listed in summary point 4 change as each parameter in each character-demography function is altered.

**6.** A wide range of joint dynamics of life history, quantitative characters and population growth can be generated in response to changes in different character-demography associations; we argue this explains the diversity of observations on the consequences of environmental change from studies of free-living populations.

**7.** The approach we describe has the potential to explain within and between species patterns in quantitative characters, life history and population dynamics.

## Introduction

Life history descriptors such as generation time and mean lifetime reproductive success, ecological variables including population growth rate and structure, and evolutionary quantities like heritability, selection differentials and phenotypic and genetic variances provide the foundations on which population biology is built. A growing number of studies report joint change in various pairs of these quantities when populations experience environmental change (Hairston *et al.* 2005). This begs the question, how are these quantities related, and should we expect them to change simultaneously when populations are perturbed? Theoretical and empirical understanding of relationships between fundamental quantities underpinning population biology would greatly extend our understanding of the dynamics of populations, life histories and quantitative characters. Such understanding could also help explain patterns in the data biologists have collected on within and between species patterns in fitness-related phenotypic characters and population dynamics. In this paper we show how a demographic model can be derived and parameterized in a way that allows many fundamental quantities in population ecology, evolutionary biology and life history theory to be calculated. Once such a model is constructed associations between these fundamental quantities can be examined through analysis of the model. Using data from a population of Soay sheep *Ovis aries* we demonstrate the ease in which such a model can be constructed, parameterized and analysed.

The fields of population ecology and evolutionary biology often appear poorly integrated although both study different aspects of the same distribution. Evolutionary questions are usually couched in terms of understanding distributions of quantitative characters and why means and variances of these distributions change with time. In contrast, population ecologists have traditionally been uninterested in the means and variances of these distributions but instead focus on how population size fluctuates. For individual characters, like body mass, the total size (or weight) of the character distribution constructed from a population at a point in time is the population size at that time. If the entire character distribution could be tracked the dynamics of the character and the dynamics of population size could be jointly investigated within a single model (Easterling, Ellner & Dixon 2000). Such a framework could also be used to gain insight into the dynamics of life history variables (Caswell 2001). Being able to simultaneously investigate the dynamics of populations, phenotypic characters, estimates of their additive genetic variances, and life history parameters, would provide a useful step in identifying linkages between population ecology, evolutionary biology and life history theory.

Unfortunately simply tracking the dynamics of a character distribution is not sufficient to link the fields of population ecology, life history and character evolution. This is because researchers in the different fields are often interested in the contribution of specific processes to observed patterns of change. The processes that evolutionary biologists are interested in include selection, phenotypic plasticity, ontogenetic development and maternal effects because these are the dominant drivers in altering the means and variances of heritable character distributions (Falconer 1960; Coulson & Tuljapurkar 2008). In contrast, population ecologists are interested in factors like density-dependence and environmental variation that often strongly influence birth and death rates, as these determine whether the size of the distribution shrinks or grows with time (Caswell 2001; Tuljapurkar 1990). Any framework that explicitly permits linkages between evolutionary biology and population ecology consequently needs to incorporate the key processes that interest each field.

Stage- and age-stage structured population models provide a powerful framework in which to investigate the dynamics of deterministic (Lefkovitch 1965); stochastic (Tuljapurkar 1990); and density- and frequency dependent populations (Caswell 2001). Structured models can also be used to identify evolutionarily stable life history strategies within the adaptive dynamics framework (Childs *et al.* 2003, 2004; Metcalf *et al.* 2008). A wide array of methods exist to analyse structured models ( Caswell 2001; Tuljapurkar 1990; Coulson *et al.* 2008). Despite the remarkable utility of structured models, they have not previously been formulated in a manner that permits the calculation of many key quantities in evolutionary biology including selection differentials and character heritability. In this paper we demonstrate that four classes of fundamental relationship need to be characterized and combined to allow the construction of structured models from which it is straightforward to calculate: (i) the distribution of modelled characters; (ii) the covariance between parent and offspring characters that is often interpreted as an estimate of additive genetic variance; (iii) the biometric heritability of the character (Jacquard 1983) calculated from the parent-offspring phenotypic covariance; (iv) terms in the age-structured Price equation including selection differentials; (v) the strength of selection on characters via lifetime reproductive success; and (vi) descriptors of the life history including generation length. In addition, the structured models we construct can be analysed using existing methods in population ecology and adaptive dynamics. Because a range of quantities of interest to population ecologists, life history theorists and evolutionary ecologists can be calculated from a single model we can analyse the model to gain insight into how the different quantities are associated. We demonstrate this using body mass and life history data from a long-term, individual-based study of Soay sheep (Clutton-Brock & Pemberton 2004). We demonstrate that the joint dynamics of quantitative characters, life history descriptors and populations can take a very wide variety of forms, and that predictions of the population consequences of environmental change can only be understood through investigation of the consequences of perturbation to the four classes of character-demography functions described above.

## Materials and methods

### Theory

Our aim in this section is to develop general theory linking integral projection models (IPMs), the Price equation, generation length and (biometric) heritability estimates (Jacquard 1983) from mother-daughter regressions. In the derivations below we allow character-demography functions to vary with time. In the empirical example that follows we keep things deliberately simple and parameterize a model for a constant environment. We work with number density distributions that describe the number of individuals within an age class with respect to character values. The area under this distribution is the number of individuals within the age class. We refer to this area as the ‘size’ of the distribution. The sum of the sizes of these distributions across all age classes is the population size.

#### Integral projection models

Age-stage-structured matrix models provide a general mathematical description (based on accounting identities) of the dynamics of population size and structure (Lefkovitch 1965). Both age and stage (or stages in the multivariate cases) are discrete. For continuous characters like body size, stage classes are constructed by binning characters into discrete stage classes. If age-specific transition rates between stages are known, the population growth rate and change in stage structure over a time step is exactly described. These transition rates are: (i) stage-specific survival; (ii) transition rates of survivors among stage classes; (iii) stage-specific fertility rates; and (iv) the stage classes into which offspring born to parents in a specific age-stage-class are recruited (defined here as reproductive allocation). All of these rates may vary with age as well as stage, and in stochastic models they may also vary with time (Tuljapurkar 1990; Ellner & Rees 2006).

The IPMs are built on functions that describe the associations between a character (or characters in the multivariate case) and survival, fertility, development of the character among survivors and the probability density distribution of offspring character values given parental characters (Easterling *et al.* 2000). In populations where dispersal rates can be ignored these are the four fundamental relationships connecting characters to demographic rates; they can vary with age and in variable environments with time (Ellner & Rees 2006). Relationships between a character and immigration and emigrations rates need to be considered in cases where dispersal rates cannot be ignored. IPMs are models that describe how number-density is added to, removed from and transformed within a uni- or multivariate character number density distribution. IPMs can accommodate both continuous and discrete traits (Ellner & Rees 2006) and are consequently mathematically more general than matrix models, although results for IPMs carry over naturally to matrix models (Easterling *et al.* 2000; Ellner & Rees 2006). The use of discrete time requires that age be counted in discrete intervals.

Assume (1) that a population is sufficiently large so demographic stochasticity can be ignored and (2) that relationships exist between a character *z* and survival *S*(*a*,*t*,*z*′), fertility *R*(*a*,*t*,*z*′), ontogenetic development of the character among survivors *G*(*a*,*t*,*z* | *z*′), and offspring character values *D*(*a*,*t*,*z* | *z*′) within each age class *a* and at each time *t*. Additionally, assume that viability selection occurs before ontogenetic development among survivors, and fertility selection (conception) occurs before reproductive allocation determines offspring character values. Models could be formulated such that growth occurs before survival, fertility, and reproductive allocation but such models are not discussed further here. Denote the number density of individuals at age *a* and character value *n*(*a*,*t*,*z*). The dynamics of this number density distribution from *t* to *t* + 1 can be written, 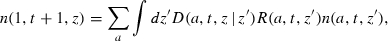
eqn 1a
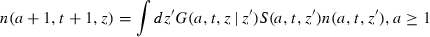
eqn 1b
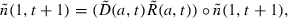
eqn 1c
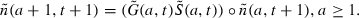
eqn 1d

Definitions of variables are provided in Table 1. Recruitment, or fertility, is defined as the number of offspring born between *t* and *t* + 1 that survive to *t* + 1. Eqn (1a) gives the number density distribution of character values among recruits to be added to the population at time *t* + 1 as a function of parental character values at time *t*. The number density distribution of offspring character values produced by each age-class is generated in two steps: a recruitment function *R*(*a*,*t*,*z*′) produces a number density distribution of parental character values that is then transformed into the number density distribution of offspring character values by the probability density function *D*(*a*,*t*,*z* | *z*′). The integral is taken over all parental character values. To obtain the population level number density distribution of newborns, we sum the age-specific number density distributions of offspring characters across all ages. As well as contributing to the offspring number density distribution, each *n*(*a*,*t*,*z*′) will produce the distribution *n*(*a* + 1,*t* + 1,*z*). Eqn (1b) describes how first a survival function *S*(*a*,*t*,*z*′) removes number density from *n*(*a*,*t*,*z*′) before a probability density function *G*(*a*,*t*,*z* | *z*′) describes how ontogenetic development transforms density among survivors.

**Table 1:** Definition of variables used in the text

Parameter	Definition
*a*	Age
*t*	Time
*z*,*z*′	Character value
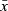	The population mean of variable *x*
*σ*^2^(*x*)	Population variance of *x*
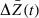	Change in character mean between *t* and *t*+1: 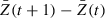
Δ*σ*^2^(*Z*(*t*))	Change in the variance of the character between *t* and *t*+1
*w*(*t*)	Mean fitness defined as the sum of mean survival and mean recruitment: 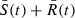
*λ*	Predicted mean fitness at equilibrium population structure
*p*(*a*,*t*)	Proportion of the population in age-class *a* at time *t*
*n*(*a*,*t*,*z*),**n**(*a*,*t*)	Continuous, discrete distribution of character values in age-class *a* at time *t*
*S*(*a*,*t*,*z*),**S**(*a*,*t*)	Continuous function, matrix, describing expected survival
*R*(*a*,*t*,*z*),**R**(*a*,*t*)	Continuous function, matrix, describing expected recruitment
*G*(*a*,*t*,*z*|*z*′),**G**(*a*,*t*)	Continuous function, matrix, describing ontogenetic development kernel
*D*(*a*,*t*,*z*|*z*′),**D**(*a*,*t*)	Continuous function, matrix, describing the reproductive allocation kernel
**Γ**,**Ψ**	Ageing matrices
**z**	Vector of midpoint character values for each age-character class
*T*	Generation time
*h*^2^	Character heritability
*V*_*a*_	Additive genetic variance of the character
*M*(*a*,*t* + *a* − 1,*z*| *z*′)	Density of offspring with character values *z* produced by parents with character value *z*′ when they were aged 1 at time *t*
*M*_*L*_	Lifetime reproductive success
*N*(*t*)	Female population size in year *t*

Eqns (1a) and (1b) describe the dynamics of a continuous character, *z*. However, it is useful to approximate IPMs in discrete matrix form to aid their analysis (Easterling *et al.* 2000). When approximated in this way we can write the kernels *D*(*a*,*t*,*z* | *z*′) and *R*(*a*,*t*,*z* | *z*′) and the functions *S*(*a*,*t*,*z*′) and *R*(*a*,*t*,*z*′) as matrices (see ‘Numerical Implementation’ below). Matrices are denoted with boldface font: for example **G**(*a*,*t*). Integral operators provide a powerful notation that covers both kernels and their matrix approximations. We denote integral operators using tildes, for example 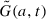. For a continuous character the integral operator is a kernel; for a discrete character the integral operator is a matrix. Eqn (1c) rewrites (1a) in integral operator notation; eqn (1d) similarly rewrites (1b). These integral operators are similar to those used in standard IPM theory (Ellner & Rees 2006) but in our development it is vital to keep separate the effects of survival (in 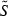), ontogenetic change (in 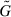), recruitment (in 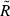) and reproductive allocation (in 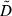) as this allows calculation of selection differentials and the biometric heritability of the character. In Table 2 we describe key number density distributions using each notation.

**Table 2:** Description of distributions and moments of distributions in continuous and discretized forms

#	Continuous	Discrete	Description
i	*n*(*a*,*t*,*z*)	**n**(*t*)	Character distribution at *t*
ii	*S*(*a*,*t*,*z*)*n*(*a*,*t*,*z*)	**S**(*t*)**n**(*t*)	Character distribution after viability selection
iii	*R*(*a*,*t*,*z*)*n*(*a*,*t*,*z*)	**R**(*t*)**n**(*t*)	Character distribution after fertility selection
iv	∫d*z*′*G*(*t*,*a*,*z*′)*S*(*t*,*a*,*z*′)*n*(*a*,*t*,*z*′)	**G**(*t*)**S**(*t*)**n**(*t*)	Character distribution after ontogenetic development
v	∫d*z*′*D*(*t*,*a*,*z*|*z*′)*R*(*t*,*a*,*z*′)*n*(*a*,*t*,*z*′)	**D**(*t*)**R**(*t*)**n**(*t*)	Character distribution after reproductive allocation
vi	*n*(*a*,*t* + 1,*z*)	**n**(*t* + 1)	Character distribution at *t* + 1
	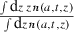	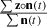	Mean of the distribution of *z*
	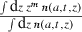	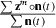	*m*th moment of the distribution of *z*

The IPMs and their matrix approximations can be used to predict population size and structure one time step ahead. IPMs also predict change in means and variances of the character number density distribution over a time step as a function of selection and other processes captured by the age-structured Price equation (Coulson & Tuljapurkar 2008). In stochastic environments the fundamental functions used to construct IPMs vary with time, and the population structure and population growth rate change from one time step to the next. However, the population converges to a stationary number density distribution of population structures and growth rates (a stochastic equilibrium) (Tuljapurkar 1990). Means and variances of the character number density distribution, as well as the population growth rate and structure, converge to equilibrium values in deterministic models, and to a stationary distribution in stochastic models.

#### From IPMs to characters and Price

The eqns (1a) and (1b) have been used to study population dynamics and the evolution of optimal character values (Childs *et al.* 2003). In this section we are interested in character dynamics rather than population numbers, and we first show that the same equations provide the tools for tracking moments of character number density distributions. The mean trait value 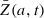 among individuals of age *a* at time *t* is just 
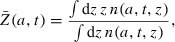
eqn 2 an equation that applies also if the character is vector-valued – that is multivariate. The *m*th moment of the character value (if scalar, or of a component, if vector) is 
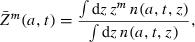
eqn 3 and the variance is then 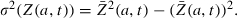


If *z*_1_,*z*_2_ are the components of a vector valued phenotypic character, we can track the phenotypic covariance via the joint moments 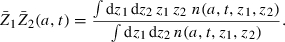
eqn 4

From here on we focus on a scalar character but the analyses extend to vector-valued characters along the lines of the above equation.

As with character values, we can compute the averages of survival rates, 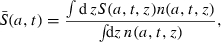 and similarly averages of ontogenetic change, fertility, and so on. Even more usefully, we can compute covariances between fitness components and characters. Thus for survival rate and character value, 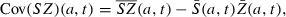
eqn 5

Readers familiar with the analysis of selection on characters will recognize the covariance in (5) as the selection coefficient on the character due to differential survival among individuals aged *a* at time *t*. Clearly, the dynamics in (1a) and (1b) make it possible to track selection acting via survival, growth, reproduction and so on. They are therefore a direct link to the fundamental frameworks used to understand the dynamics of phenotypic characters – the Price equation (Price 1970) and the breeders equation (Falconer 1960). These frameworks are both formed in terms of selection differentials.

The age-structured Price equation describes change in the population level mean of a number density character distribution between time *t* and *t* + 1 (Coulson & Tuljapurkar 2008). An equation for change in the variance has also been derived (S. Tuljapurkar & T. Coulson, unpublished). Terms in the Price equation describe how survival, recruitment, ontogenetic development and reproductive allocation alter the mean of number density distributions within and between each age-class (Table 2). In addition to these contributions we need two additional quantities to write a form of the age-structured Price equation (Coulson & Tuljapurkar 2008). First, the normalized density at character value *z* of individuals at age *a* at time *t* is 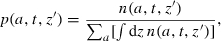 and the fraction of individuals at age *a* at time *t* is 
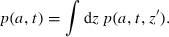


Second, the growth rate of the population between *t* and *t* + 1 is 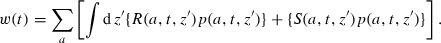
eqn 6

In the following equations we denote means and variances of the number density distributions across all ages as 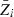 and *σ*^2^(*Z*_*i*_) for *i* = i,…,iv (Table 2). Change in the mean, 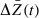 (Coulson & Tuljapurkar 2008), and change in the variance, Δ*σ*^2^(*Z*(*t*)) (S. Tuljapurkar & T. Coulson, unpublished), of the character number density distribution can then be written, 
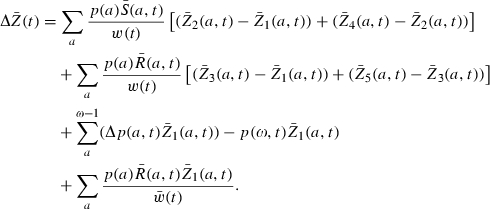
eqn 7 and 
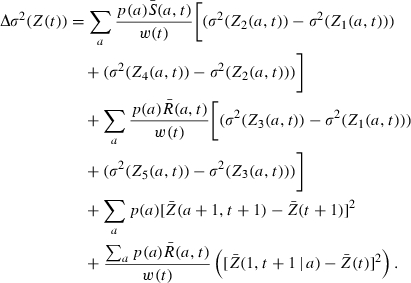
eqn 8

The terms in square brackets in the first two rows of both equations describe differences in the mean (7) and variance (8) between pairs of character number density distributions (Table 2). In both cases the first row describes contributions via survival and ontogenetic development while the second row captures contributions via recruitment and reproductive allocation. The remaining terms deal with contributions to change via fluctuations in the age-structure. In what follows we focus on change in the mean (7), although the interpretation is the same for the variance and for any higher central moment of interest.

The first term on the right in the top row of (7) describes how viability selection shifts the character mean; it is a viability selection differential. The second term on the right of (7) in the top row describes the average rate of ontogenetic development of the character among survivors. In the second row of (7) the first term in square brackets is a fertility selection differential, while the second term describes the average difference between offspring and parental character values (reproductive allocation). The terms outside square brackets in the first two rows of (7) provide the demographic weights needed to average the terms in square brackets across age-classes. These first two rows in (7) describe how within age-class processes change the mean value of the character. The bottom row describes how differences between age-classes alter the character number density distribution. In (7) the first term in the bottom row on the right describes how differences in mean survival rates between age-classes alters the character number density distribution, while the second term describes how differences in reproductive rates between age-classes contribute to change. These terms will be non-zero in populations at equilibrium if there are survival and fertility differences between age-classes that are independent of the character.

#### Calculating life history and quantitative genetic quantities

Our model can also be used to calculate life history descriptors like generation time and net reproductive rate, as well as an estimate of the character heritability and selection on the character via lifetime reproductive success. To calculate these quantities we first show how to track the performance of cohorts in terms of survivorship and fertility. We use our integral operator notation.

We track cohort dynamics by iterating eqn (1d). Between ages 1 and 2, changes in a cohort are described by the integral operator 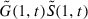 as in (1c) and (1d). Between ages 2 and 3, the corresponding operator is 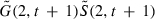, and so on. String these together to obtain 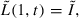
eqn 9
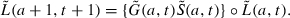
eqn 10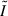 is the identity kernel (continuous formulation) or identity matrix (discrete formulation), and 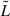 is a kernel (continuous formulation) or matrix (discrete formulation) describing survivorship. In the continuous notation of Easterling *et al.* (2000) the iteration in (10) is as follows, 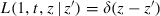
eqn 11
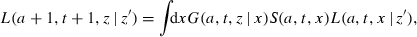
eqn 12 where *δ*(*z* − *z*′) is the Dirac delta function. In discrete space the Dirac delta function is the Kronecker delta.

The expected number density of offspring with character value *z* produced at age *a* by a parent born at time *t* with a character value *z*′ is denoted *M*(*a*,*t* + *a* − 1,*z* | *z*^′^) and is given by 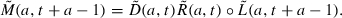
eqn 13

To find the lifetime reproduction 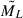 of a parent born at time *t* with character value *z*′, we add together offspring produced at all ages through some (possibly large) maximum age *A*, 
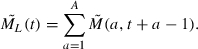
eqn 14

A cohort born at *t* with a character number density distribution 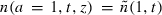 produces at age *a* an offspring number density distribution 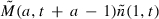, and a lifetime offspring distribution 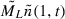.

We can use these equations to calculate a number of life history quantities. Such calculations make sense in a constant, density-independent environment, when rates are time-independent. In our model, age-dependence complicates the known methods (Ellner & Rees 2006). We define generation time *T* by a frequently used identity (Caswell 2001), which is tautological but may be useful. We calculate generation time *T* by the identity *R*_0_ = e^*rT*^ where *r* is the asymptotic growth rate and *R*_0_ is the net reproductive rate. *R*_0_ is the dominant eigenvalue of the operator *M*_*L*_ in (14): recall that this operator describes lifetime reproduction. The asymptotic growth rate describes the growth rate when the population has a stationary age and character density distribution. In the latter, denote the character number density distribution of newborns (age class 1) by 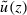; recall the integral operators 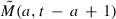 in eqn (13) that describe the number density of offspring of a cohort when that cohort reaches age *a*. In the stationary state these operators do not depend on time so we can write them simply as 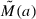. Then *r* is the solution to the integral equation (U. Steiner, S. Tuljapurkar & T. Coulson, unpublished) 
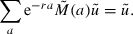
eqn 15

In practice no one is going to solve these integral equations. Instead we use a discrete matrix approximation and turn the integrals into sums, as we illustrate in the next section.

We now turn to the breeder's equation. The breeder's equation has been widely used to understand phenotypic change of heritable characters (Bulmer 1980; Lande & Arnold 1983). Specifically it describes the response to selection defined as the per generation change in the mean of the breeding value distribution. A breeding value of a character describes the additive genetic worth of a parent for that character.

The breeders equation, in the univariate form considered here, contains two terms – a selection differential between the character and lifetime reproductive success (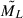) and a character heritability, *h*^2^. The heritability is the ratio of the additive genetic variance *V*_*a*_ to the phenotypic variance *σ*^2^(*Z*). Heritabilities and additive genetic variances can be estimated in many ways. The classic biometric approach we use here is through a regression of daughter character values measured at age *a* against maternal character values also at age *a*. Twice the slope of the regression line is the character heritability (Falconer 1960). This approach is entirely statistical; hence the use of the term biometric heritability. As discussed by Jacquard (1983) the connection between this biometric heritability and any underlying genetic variation is not simple, even though it is often assumed to be so (see discussions in Willis, Coyne & Kirkpatrick 1991; Kruuk 2004).

To estimate the biometric heritability of body mass measured at age 1, we need to consider parents born into different size classes and track the number density distribution of the stage classes of offspring born to these parents at each age in the life course. To do this we start with a cohort of newborns who progress through the life cycle to become parents. This cohort of newborns is described by a number density over character values, 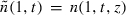, which we iterate forwards to track the number density distribution of offspring produced. We consider all offspring produced over a lifetime. From eqn (14) we see that the joint number density distribution of offspring character value *x* and parental character value *y* must be proportional to 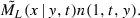


In a one sex model, the regression of offspring trait value *Z*^*o*^ on parental trait value *Z*^*p*^ has a slope that equals half the heritability, 
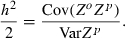
eqn 16

From the joint number density distribution we have 
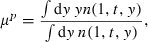
eqn 17
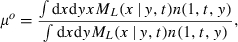
eqn 18
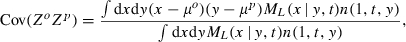
eqn 19
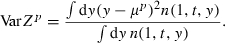
eqn 20

The first two equations above yield the mean character values of parents and offspring, respectively. The third, eqn (19), is the important new relationship here and shows that the character number density distributions from the model can be used to compute the parent-offspring covariance that is the key to determining biometric heritability. Eqn (20) yields the variance among parents. We can now compute half the heritability *h*^2^ using eqn (16).

It is important to note that the value of *h*^2^ does not depend on the number of parents that we start with as newborns, only on their character distribution [because the number cancels out of the ratio in (16)]. Several aspects of biometric heritability can be explored using our analysis. First, we can compare the equilibrium heritability obtained by starting with a stable character density distribution of newborns (i.e. 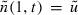) with time-dependent values for cohorts who are observed over their reproductive lives. Such a comparison will illuminate the effects of environmental change on life history transitions. Second, we could compute age-dependent biometric heritabilities at each age *a* by repeating the covariance calculation in eqn (19) but considering only offspring produced at age *a*– we will do this in future work. This would allow us to examine the effects of age, a variable that is often factored out of the usual models for estimating heritability by treating age as a covariate.

The equations above show how a wide range of population biology quantities can be calculated from IPMs. Implementation of the model requires us to approximate the model in matrix form (Easterling *et al.* 2000). In the next section we explain how this is done.

#### Numerical implementation

The continuous character-demography functions on which IPMs are built are identified through statistical analyses (Easterling *et al.* 2000). Predicted values from these continuous functions can be calculated for very small-width discrete bins, and these values used to construct a high dimensional transition matrix. An IPM will only accurately capture the dynamics of a population and a character distribution if the statistical functions used to construct the model accurately capture observation. As with any statistical analysis, the identification of accurate and appropriate functions requires good data and biological knowledge of the system under study. Let us assume that functions have been identified. How would our IPM [(1a) and (1b)] look in matrix form?

To write our discretized age-stage IPM as one large matrix (Lebreton 1996) requires a note on notation. The age-specific continuous number density distributions 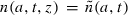 are combined into one vector **n**(*t*). The *i*th element of this vector represents the number of individuals in age-class *a* and character class *j*. Each possible character class is included within each age even if no individual of age *a* can have that value. For example, it may be impossible for a new recruit to have an average adult body mass. However, there is an element in **n**(*t*) at this impossible character value but this element will always be zero. If we have nine age-classes and 100 character classes **n**(*t*) will consequently be of length 900, with elements 1–100 representing age class 1 (**n**(1,*t*)), elements 101–200 representing age class 2 (**n**(2,*t*)) etc.

A set of square matrices is used to iterate **n**(*t*) to **n**(*t* + 1) with matrix elements equal to predicted values of *S*(*a*,*t*,*z*), *R*(*a*,*t*,*z*) (both diagonal matrices), *G*(*a*,*t*,*z* | *z*′) and *D*(*a*,*t*,*z* | *z*′) calculated at the mid point value of each stage class. We also define a vector **z** consisting of these mid-point values of each stage-class (Easterling *et al.* 2000). In our age-character model **z** consists of the list of mid-point values for phenotypic classes repeated by the number of age-classes. The vector **z** is required to calculate quantities describing character dynamics (Table 2).

An age-structured IPM is now approximated in matrix form as, 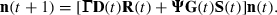
eqn 21

Each of the matrices **S**(*t*), **R**(*t*), **G**(*t*), **D**(*t*), **Ψ** and **Γ** are square ‘block’ matrices consisting of an array of age-specific matrices defined above. An age-specific sub-matrix of this large matrix is described with indices (*a*,*t*). The **S**(*t*) and **R**(*t*) matrices are diagonal describing survival and recruitment rates of individuals in each age-character class – they are discretized versions of *S*(*a*,*t*,*z*′) and *R*(*a*,*t*,*z*′). Each sub-matrix **G**(*a*,*t*) describes transition rates between stage classes within an age-class among survivors, except it does not yet age the survivors by 1 year (see below). Each sub-matrix **D**(*a*,*t*) describes the transition rate from maternal stage to offspring stage, except that it does not yet place offspring into the age-class of new recruits. See the online appendix for a figure displaying the form of these matrices.

The matrices **Γ** and **Ψ** describe age transitions. **Γ** moves offspring out of the maternal age class into the new recruit class (aged 1) – the top row of sub-matrices of **Γ** are all identity matrices while all other sub-matrices contain only zeros. **Ψ** acts in a similar manner to **Γ** but ages survivors. **Ψ** and **Γ** are time invariant. The functions *D*(*a*,*t*,*z* | *z*′) are approximated by **ΓD**(*t*), while the functions *G*(*a*,*t*,*z* | *z*′) are approximated by **ΨG**(*t*). We will report quantities at equilibrium, so now drop the index *t*.

The dominant eigenvalue of (21), *λ*, is the population growth rate (Lebreton 1996), and the left and right eigenvectors associated with *λ* are respectively the reproductive value and stable age-character distribution. Our integral operator notation demonstrates it is straightforward to rewrite our equations for the age-structured Price equation and the breeders equation in matrix form. Having calculated quantities in these equations we next explore how they are related to one another. We first construct a matrix and calculate key quantities at equilibrium. Next, we independently perturb parameter values in the character-demography functions and examine how each perturbation alters each of the quantities. This is a form of sensitivity analysis (Caswell 2001). We perturb function parameters rather than specific matrix elements because we believe that environmental variation and evolution will change these functions. We chose not to centre functions prior to perturbation because most published character-demography functions are not centred.

### Data

The population of Soay sheep *Ovis aries* living in the 250 ha Village Bay catchment of the Island of Hirta in the St. Kilda archipelago, Scotland, has been studied in detail since 1985 (Clutton-Brock & Pemberton 2004). There are no sheep predators on the island, and no interspecific competition for forage from other large herbivores meaning the population is only food limited. Each spring newborn individuals are caught and uniquely marked with ear tags within hours of birth. Mortality tends to occur during the winter months. Regular mortality searches during this period result in the majority of carcasses being found, normally within a day of death. Since 1986, each spring, summer and autumn, 10 censuses of the population are attempted. Over 95% of individuals seen in these censuses are identified – the unidentified individuals tend to be transients seen on the edge of the study area (Coulson, Albon, Pilkington & Clutton-Brock 1999). The birth, death and census data are used to provide a list of which individuals are living permanently within the study population each August. The population size in each year is consequently known accurately. The population exhibits periodic crashes when up to 70% of the population can die (Coulson *et al.* 2001). Maternity is inferred from observations of birth or suckling. Each August a team catches as many individuals as possible – on average 50% of the resident population. Any unmarked individuals that are caught are marked. Each time an individual is caught blood samples, faecal samples and a range of phenotypic data including body mass are collected. Observed body mass means and variances both within and across age-classes are given in Table 3.

**Table 3:** Observed and predicted quantities. Predictions obtained assuming equilibrium age-character structure

Quantity	Observed	Predicted	Quantity	Observed	Predicted
*λ*	1·05^a^	1·03	*T*	4·09^b^	5·85
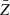	19·15	19·30	*σ*^2^(*Z*)	28·21	28·19
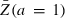	12·55	12·11	*σ*^2^(*Z*(*a* = 1))	6·17	7·07
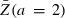	17·41	17·08	*σ*^2^(*Z*(*a* = 2))	5·92	5·91
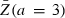	22·67	22·70	*σ*^2^(*Z*(*a* = 3))	7·66	7·01
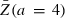	23·95	23·79	*σ*^2^(*Z*(*a* = 4))	8·03	6·75
*h*^2^	0·18	0·20	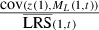	1·17^c^	1·14
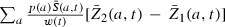	0·22^d^	0·21	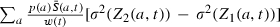	−0·41	−0·30
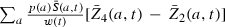	1·08	0·96	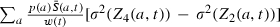	−0·05	−0·04
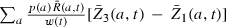	0·07	0·07	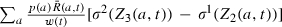	−0·05	−0·03
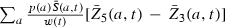	−2·33	−2·41	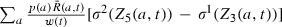	−0·50	−0·44
Other terms in (7)	1·25	1·17	other terms in (8)	1·08	0·81

a Arithmetic mean of 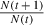.

b Calculated from the Soay sheep life table.

c Calculated from individual-based data.

d All observed values in (7) and (8) calculated from individual-based data (Coulson & Tuljapurkar 2008; S. Tuljapurkar & T. Coulson, unpublished).

We used data on life history and body masses collected from the female component of the population between 1986 and 2008. Although we focus on body mass, any morphological, physiological, genetic or behavioural character could be used in our approach. We consider four age-classes identified from previous analyses of survival rates: lambs, yearlings, prime-aged adults aged 2–6 years, and senescent individuals aged over seven (Coulson *et al.* 2008). A detailed description of data collection protocols is provided elsewhere (Clutton-Brock & Pemberton 2004).

Individual body mass, survival, fertility and offspring mass data were used to identify functions required for parameterization of integral projection models. We fitted generalized linear models with a binomial error structure to annual individual survival, reproduction, and, for those individuals that bred, whether one or two recruits were produced. We defined reproduction as whether an individual bred and produced offspring between *t* and *t* + 1 that survived to enter the population in the August after birth (*t* + 1) (Coulson *et al.* 2001). The litter size (twinning) function described the number of recruits each female produced (1 or 2). Growth rate functions were estimated by using multiple linear regression models of mass in year *t* + 1. Reproductive allocation functions were estimated through multiple linear regression of the mass at *t* + 1 of offspring produced between *t* and *t* + 1 that recruited to the population in *t* + 1. There is considerable temporal variation in demographic rates (Coulson *et al.* 2001). We fitted year class as a categorical variable in all models to correct for this variation. Obviously body mass was fitted as an independent term in all models. We fitted separate models for each of the four age classes.

The resultant character-survival functions *S*(*a*,*t*,*z*) are of the form exp (*α* + *βZ*)/(1 + exp (*α* + *βZ*)) where *α* and *β* are obtained from logistic regressions for survival. The functions *R*(*a*,*t*,*z*) are obtained by combining the reproduction and litter size (twinning) functions, both which are of the same form as the logistic models for survival. If we define the twinning functions as *φ*(*a*,*t*,*z*) and the fertility functions as *F*(*a*,*t*,*z*) then *R*(*a*,*t*,*z*) = *F*(*a*,*t*,*z*)(1 + *φ*(*a*,*t*,*z*)). To estimate growth kernels *G*(*a*,*t*,*z* | *z*′) it is necessary to combine the function describing mean body size at year *t* + 1 given body size in year *t* with a function describing the variance around these associations and scaling so that all transition rates out of an age-stage class sum to unity. The variance function is identified by regressing the squared residuals around the mean body mass function against body mass (Easterling *et al.* 2000). We found no compelling evidence for nonlinearity in these functions so used linear regressions. We define the intercept and slope of the linear regression of body mass in year *t* + 1 against body mass in year *t* as *α*_*μ*_ and *β*_*μ*_ and the intercept and slope of the variance function as *α*_*σ*_ and *β*_*σ*_. If we next define 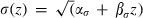 and *μ*(*z*) = *α*_*μ*_ + *β*_*μ*_*z*, the probability density function describing transition rates between *z* and *z*′ is, 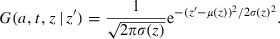
eqn 22

The same logic is used to define the *D*(*a*,*t*,*z* | *z*′) functions for each age-class. We next define the integration limits in (1a) and (1b) that also provide the smallest and largest values of **z**. The smallest observed August female sheep mass was a recruiting lamb weighing 2·9 kg and the largest was from a 34·2 kg adult. When constructing **S**(*t*), **R**(*t*), **G**(*t*) and **D**(*t*) we generated 100 phenotypic classes ranging from 0 to 37·5 kg as this provided a good approximation to the continuous functions (Easterling *et al.* 2000): decreasing bin size further had no influence on all quantities calculated to 2 decimal places. We ensured that transition probabilities out of class *j* for the kernels **D**(*a*,*t*) and **G**(*a*,*t*) summed to one by dividing each element by the sum of estimated transition probabilities. All statistical analyses and model construction and analysis were conducted in (R Development Core Team 2009). Code for constructing IPMs utilized functions provided by Ellner & Rees (2006).

For the matrix approximation of the IPM we calculated the following quantities at equilibrium (we consequently drop the index *t*): asymptotic population growth, *λ*; mean character value at the population level 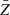 and within each of our four age classes 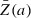 for *a* = 1,…,4; variance in character value at the population level *σ*^2^(*Z*) and within each of our four age classes *σ*^2^(*Z*(*a*)) for *a* = 1,…,4; the contributions of viability selection, fertility selection, growth among survivors, reproductive allocation and the demographic weights to 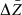 and Δ*σ*^2^(*Z*) summed over age-class (Table 3); generation length *T*; heritability of body mass *h*^2^; the selection differential between body mass as a recruit to the population and lifetime reproductive success. We compared these model predictions to the same quantities calculated from the individual-based data or with previous published estimates (Table 3).

Intercepts and slopes of each of the statistical functions used to parameterize the IPMs were independently perturbed by 1% and new IPMs and matrix approximations constructed. The direction of each perturbation was chosen so as to increase *λ*. The perturbed matrices were then used to calculate the the proportional change in the equilibrium values listed above. Because the *S*(*a*,*t*,*z*) and *R*(*a*,*t*,*z*) functions were on the logistic scale we rescaled perturbations to the same scale as the *G*(*a*,*t*,*z* | *z*′) and *D*(*a*,*t*,*z* | *z*′) functions.

## Results

**Fig. 1:**
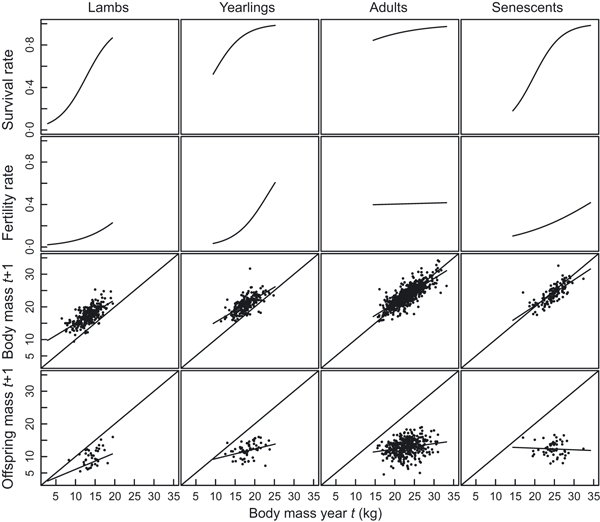
Shapes of the statistical functions between body mass and survival, fertility, mean growth rates and mean reproductive allocation within each of the four age-classes used to parameterize the integral projection model. Twinning rate functions and body-mass variance functions (see text) are not displayed. Points represent raw data; lines represent predictions from regressions including year class to correct for temporal variation. In the bottom eight plots the function *y* = *x* is also plotted.

Associations between body mass and: (i) survival; (ii) fertility; (iii) next year's body mass among survivors; and (iv) offspring body masses when they recruit to the population are displayed in Fig. 1. We do not display the body mass-twinning functions or the functions describing variances around (iii) and (iv). Parameter values for all functions are in the online appendix. Our focus in the results that follow is on the ecological and evolutionary descriptors of the population at equilibrium. We start by comparing model predictions with observations, then describe how equilibrium predictions change as model parameters are altered.

**Fig. 2:**
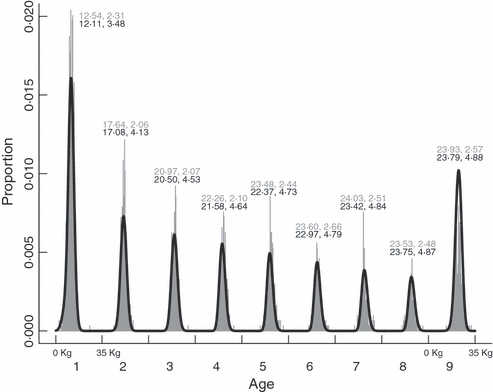
Observed and predicted age-character number density distribution. Grey bars represent the observed number density distribution; the black line represents that predicted by the model. Each pair of numbers represents mean and variance of body mass within each age. Grey numbers are the first two central moments of observed distribution, while black numbers represent the same quantities for the predicted distribution.

In general, predictions of the quantities we estimated from the matrix approximation of the IPM corresponded well with observation (Table 3). The largest mismatch is between observed and predicted generation time. This mismatch occurs because empirical estimates of generation time are affected by environmental variation (see appendix 1 in Clutton-Brock & Pemberton 2004) that contributes to cause periodic crashes in the sheep population when up to 70% of the population dies (Coulson *et al.* 2001). Our deterministic and density-independent model does not capture this variation. Our model captured age-specific mean body mass very well, but over-estimated the standard deviation of body mass in all age-classes (Fig. 2). This mismatches arises because our normal distribution describing the variance function around *G*(*a*,*t*,*z* | *z*′)eqn (22) does not exactly capture the observed transition rates. As our goal here is to illustrate the methods and their uses, not accurate prediction, we continue analysis of this model despite the mismatch in the variances.

Our estimate of the character heritability was consistent with published estimates obtained through use of the animal model (Table 3). We did not correct for common environment (including maternal) effects in our analyses but describe how such correction could be conducted in the discussion.

### Perturbation

**Fig. 3:**
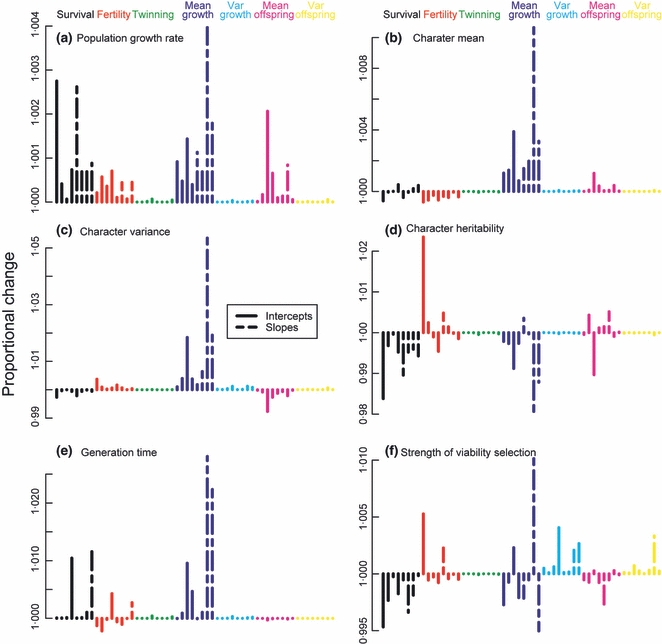
The proportional consequence of a 1% perturbation to intercepts (solid bars) and slopes (dashed bars) of the character-survival (black bars), fertility (red bars), twinning rate (green bars), mean growth rate (blue bars) and mean reproductive allocation (pink bars) functions for six quantities (a) the population growth rate *λ*, (b) the population-level mean of the character 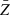, (c) the population-level variance of the character *σ*^2^(*Z*), (d) the character's heritability *h*^2^, (e) the generation length *T* and (f) the strength of viability selection calculated across the life cycle (Table 3). In addition displayed are the consequences of perturbing intercepts and slopes of the functions describing variation around the body mass-growth rate (indigo bars) and reproductive allocation (yellow bars) functions. For each parameter four bars are displayed. The first bar represents consequences of perturbing parameters in lamb functions, the remaining bars (in order) show the consequences of perturbing parameters in the yearling, prime-aged adult and senescent age-classes.

**Fig. 4:**
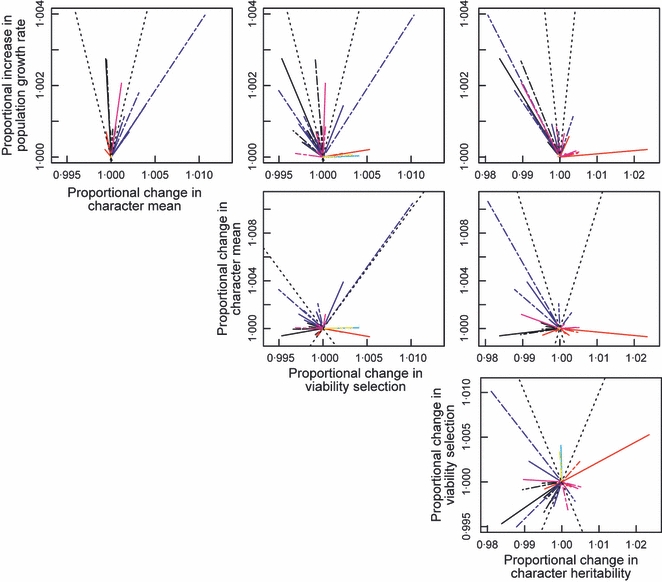
A pairs plot showing the joint consequence of perturbing parameters in the intercepts and slopes of character and demography functions on different combinations of quantities. For keys to colours and line styles see the legend to Fig. 3. Although we plot the consequences of perturbations to parameters in each age-class specific function, we have not attributed lines to age-classes. Note that these plots should be seen as correlations and not representing a direction of causation – in other words the *x* and *y* axes could be reversed without loss of meaning. The driver of changes results from perturbations to the various character-demography functions.

We now turn to the effect of perturbations on the population at equilibrium. In Fig. 3 we show how perturbations impact six quantities: the population growth rate, the mean and variance of the character number density distribution, the biometric estimate of the character heritability, the generation length and the total strength of viability selection across the life cycle. In Fig. 4 we focus on joint change in four quantities: population growth, the mean of the character, the character heritability and the strength of viability selection. Below we focus on key findings relevant to the issues we raise in the discussion; descriptions of how specific results arise can be found in the online appendix.

Population growth *λ* (also mean fitness in deterministic environments), unsurprisingly, increased with increasing survival and fertility rates, with increasing growth rates that move individuals more quickly into the fertile ages, and with increase in mean offspring size. Generation time (which we compute via growth rate *r* and net reproductive rate *R*_0_) responded differently: it was not sensitive to early survival, decreases when early fertility increased, and increased with senescent fertility and survival; these changes can be understood in terms of the offsetting increases in *r* and *R*_0_. The changes we found in the mean character value and the overall strength of viability selection are consequences of the changing age-stage structure of the population. When early survival rates increase or growth rates increase, the population structure shifts to include a higher proportion of larger older individuals; increasing early fertility has the opposite effect. A similar, but smaller, effect was observed through perturbing parameters in the mean function in the reproductive allocation kernels *D*(*a*,*t*,*z* | *z*′). For both types of kernel, increasing parameters generates larger individuals that increases survival and fertility rates and consequently changes both population growth and age-stage structure.

Estimates of heritability were also influenced by the same functions as was population growth, although the direction of change was often different. To understand these effects note that the covariance between parent and offspring can change with age (along with the kernel *D*). Thus the covariance will be age-specific, and the overall heritability that we estimate is an average of age-specific covariances. Increased survival rates imply that an individual's total reproductive output is more dispersed with respect to its age, and as the slope of the mean growth rate functions tend to unity the total reproductive output becomes dispersed over a wide range of sizes. The result is to lower the overall parent-offspring covariance and reduce heritability. Increasing early fertility, on the other hand, tends to reduce the age-dispersion of lifetime reproduction, and thus increases heritability. It is noteworthy that the parent-offspring covariance and heritability are most sensitive to perturbations of the growth rates of parents between birth and ages at reproduction (Fig. 3). Growth rates in the sheep are strongly influenced by temporal environmental variation (Clutton-Brock & Pemberton 2004), which suggests more accurate estimates of heritability could be obtained if environmental variation in growth rates were corrected for in statistical analyses.

Perturbing parameters in the variance functions used to construct the transition kernels 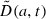 and 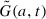 had trivially small effects on all quantities with the exception of viability selection, where effects were just small. Perturbing these parameters in the 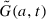 kernel influences rates of stasis within a character class: an increase in the intercept decreases stasis. If individuals are likely to remain within a character class throughout life (high stasis), this is interpreted as persistent individual differences. Increasing the slope of the function decreases rates of stasis amongst individuals with larger character values (i.e. there is greater stasis at some character values than others).

Figure 3 demonstrates that a small perturbation to a function parameter can act to increase estimates of some quantities while simultaneously decreasing others. Patterns of change in pairs of quantities are not always in the same direction when different parts of the demography are perturbed. Figure 4 shows how a wide range of different joint outcomes can be observed depending on which functions, and parameters within functions, are altered. Nearly all types of joint dynamic are possible: in nearly all cases an increase in one quantity can go hand-in-hand with either a decrease or an increase in another quantity. This suggests that environmental change can theoretically generate a very wide range of eco-evolutionary dynamics.

## Discussion

In this paper we first demonstrate how many key quantitative genetic quantities including biometric heritability, selection differentials and terms in the age-structured Price equation can be calculated from age-stage-structured models. Age-stage-structured models are a powerful tool already widely used in life history theory and population ecology. Demonstrating how they can be used to calculate quantitative genetic quantities allows life history and quantitative character evolution and population dynamics to be examined within the same formal framework. Application of this framework to Soay sheep demonstrates the ease with which models can be constructed and analysed. As well as providing specific insight to the Soay sheep system, our approach reveals that change to any one aspect of the character-demography functions in the model can simultaneously influence several characteristics of the population. Perturbation of different character-demography functions can generate a wide range of eco-evolutionary dynamics.

### Heritability estimates

The heritability we calculate is based on the phenotypic covariance between parents and their offspring. We calculate the parent-offspring phenotypic correlation by following the fate of each stage-class within a cohort of new recruits to the population, recording the number density distribution of offspring trait values produced by this cohort at each age. The 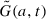 operator describes mixing: how individual stage classes at age *a* at time *t* can contribute density to multiple stage classes at age *a* + 1 at time *t* + 1. The 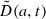 operator describes another kind of mixing: how individual stage classes among age *a* mothers contribute density to multiple stage classes among age 1 offspring at time *t* + 1.

The speed at which mixing happens within the IPM influences the estimates of the heritability of the character. The degree of mixing is determined by both the slope and variation around the ontogenetic and reproductive allocation kernels (Fig. 1). The closer the mean slope of these kernels is to zero and the greater the variance around these slopes, the faster the degree of mixing. If mixing is very rapid then the heritability will be small. This occurs because any character value for a cohort produces a similar number of offspring across all possible offspring character values. If mixing occurs slowly then the estimate of the heritability will be high. In matrix terminology mixing between stages means a matrix is ergodic and irreducible: trajectories from one stage-class to all others are possible, even if it takes many time steps for descendants of an individual born into stage-class *j* to be represented in all possible stage classes.

What does this mixing imply for a heritable quantitative character? Because complex quantitative characters are influenced by large numbers of loci and environmental variation, a genotype at a locus can be present in individuals with a wide range of character values. This is because, in non-haploid systems, if large numbers of multi-allelic loci influence the character there will be a very large number of genes that influence the trait – perhaps each multi-locus genotype within a population will be unique. Given that genetic background experienced by a genotype at a locus (Schuetz *et al.* 2000), epistasis (Goodnight 1988), the non-genetic developmental environment (Parker & Begon 1986) and indirect genetic effects (Wolf *et al.* 1998) all influence how a genotype at a locus contributes to the development of quantitative characters, we expect the genotype-phenotype map to be one-to-many rather than one-to-one. A consequence of a single genotype potentially being expressed in multiple different character values is that predictions of evolutionary change using quantitative genetic parameters like the biometric heritability for characters determined by large numbers of genes are unlikely to provide predictions of evolutionary change beyond a generation (see also Lande & Arnold 1983). The additive genetic variance estimated from statistical methods is likely accurate for one period only, but long-term evolutionary change depends upon the genetic details, including the effects of recombination (Turelli & Barton 1994).

Our heritability estimates may not solely reflect additive genetic variance of the character. We estimate heritabilities using a form of parent-offspring regression. Our estimates of heritability will include contributions from maternal effects, non-additive genetic variance and other components of variance that can be corrected for using the animal model (Bulmer 1980). It would prove useful to be able assess how each of these processes contribute to the quantities we calculate within our framework. This could be achieved by comparing results from IPMs parameterized with statistical functions designed to correct for specific components of the variance with those parameterized from statistical models that do not make such corrections. If statistical approaches appropriately correct for all sources of variance that can influence mother-offspring phenotypic covariances – for example permanent environment effects and the additive genetic variance – we would expect the slope in the reproductive allocation functions (Fig. 1) estimated from these regressions to be flat. Although we did not correct for permanent maternal effects in our estimates of heritability it is noteworthy that our estimate matched remarkably well with those obtained from application of the animal model. This is perhaps not surprising: despite the advantages the animal model offers over parent-offspring regression it is not unusual for heritability estimates obtained using the two approaches to be of similar magnitude (Kruuk 2004; Akesson *et al.* 2008).

### Model performance and joint dynamics

In general predictions from our IPM matched observed estimates reasonably closely. Our statistically identified character-demography functions do a good job of capturing the raw association in Fig. 1. They do not attempt to correct for processes like density dependence and environmental drivers that generate these patterns. If correction for a specific process, like density-dependence, in the statistical analyses used to parameterize an IPM results in a substantial deterioration in the correspondence between observation and prediction then it can be inferred that this process is influential in generating observed dynamics. Similar logic was used by Coulson *et al.* (2008) to assess the contribution of density-dependence and environmental variation to the population dynamics of the Soay sheep system.

We next examined how perturbing model parameters influenced the quantities we calculated. Perturbing any one function parameter simultaneously influenced estimates of all quantities we examined. This finding is consistent with field observations showing that populations respond in contrasting ways to environmental change depending on which character-demography association is altered. For example, a decrease in body mass occurred with a concurrent decrease in population size in Bighorn sheep (Coltman, Festa-Bianchet, Jorgenson & Strobeck 2002) when adult body-mass survival functions were altered by selective hunting, while a decrease in body mass with a simultaneous increase in population size was observed in Soay sheep when environmental change increased body mass-survival functions and reduced body mass-growth functions (Ozgul *et al.* 2009).

Our model also provides unprecedented insight into linkages between the fundamental parameters of population biology. Perturbations of any part of a life cycle has downstream effects within and across generations (Prout & McChesney 1985; Roach & Wulff 1987) with multiple ecological and evolutionary consequences (Fig. 3). In order to understand the consequences of perturbing a parameter it is necessary to investigate how it alters the transition rates between character stages across the entire life cycle. Our analyses demonstrate how complex the consequences of a single perturbation can be and support the argument that an understanding of ecological and evolutionary dynamics requires examination of character-demography associations across the entire life cycle (see also Prout 1971; Caswell 2001; Tuljapurkar 1990; Charlesworth 1994; Benton & Grant 2000; Ellner & Rees 2006). For example, in the Soay sheep, we find that increasing fertility rates early in life would lead to both a decrease in mean size and a decrease in selection for larger size. It would be impossible to predict this response by considering only a single character and a single fitness component, or only a scalar measure of individual lifetime fitness. Statistical analysis of single character-demography associations is certainly valuable, but predictions must consider all other character-demography associations in the life cycle.

### Fundamental functions

A distribution can be modified by removing density from it, adding density to it, and moving density within it. In the absence of dispersal, the four functions we describe (character-survival, character-fertility, character-ontogenetic development and character-reproductive allocation) are the only biological processes that change density. All the quantities we calculate, and indeed all meaningful quantities that can be calculated within population biology, derive from these functions. We believe that continued development of structured models will prove fruitful in understanding how fundamental parameters in population biology are influenced by processes as diverse as maternal effects and persistent individuals differences, environmental and demographic stochasticity, density and frequency dependence, genotype-by-environment interactions and phenotypic plasticity.

## Conclusions

Our approach (and all IPMs) require the statistical analysis of data routinely collected by field and laboratory biologists to parameterize character-demography associations – there are numerous systems where sufficient data exist (Jones *et al.* 2008). In addition to developing models for other systems, possible extensions include: the incorporation of density-dependence, environmental stochasticity, multivariate characters, and explicit genotype-phenotype maps; developing models for continuous time and for two sexes; examination of how incorporation of random effects in statistical models influence predictions; integration with adaptive dynamics methods; and derivations of analytical sensitivities. These extensions are achievable with approaches already developed for IPMs and matrices (Charlesworth 1994; Tuljapurkar, Horvitz & Pascarella 2003; Tuljapurkar & Haridas 2006; Caswell 2009; Rees & Ellner 2009).

Change in a character, or population size within a population of a single species, can have effects elsewhere within the ecosystem through altering patterns of interspecific competition, rates of predation and causing trophic cascades (Fortin *et al.* 2005). So although our focus here has been limited to a single population, insight into multi-species systems could be obtained through investigating: (i) when an alteration to a character-demography association within a species has knock on effects; and (ii) biotic and abiotic factors that generate change in character-demography associations. So although we focus on a single species, the processes we describe are relevant for change in communities and ecosystems. The work we develop here, which extends the framework proposed by Coulson *et al.* (2006) and builds from the important insights of Easterling *et al.* (2000); Ellner & Rees (2006) and Lebreton (1996), shows how demography is central to all endeavours in population biology. To paraphrase Metcalf & Pavard (2007), all population biologists should be demographers.

## Supporting Information

**Appendix S1.** Additional interpretation of results displayed in Figure 3.

**Fig. S1.** Structure of the **S**(*t*), **R**(*t*), **G**(*t*), **D**(t), **Ψ** and **Γ** matrices. Grey cells represent elements that can be non-zero while elements in white are always 0. The square highlighted with the dotted lines represent matrix elements associated with one age-class (age 2).

As a service to our authors and readers, this journal provides supporting information supplied by the authors. Such materials may be re-organized for online delivery, but are not copy-edited or typeset. Technical support issues arising from supporting information (other than missing files) should be addressed to the authors.

## References

[b1] Akesson M, Bensch S, Hasselquist D, Tarka M, Hansson B (2008). Estimating heritabilities and genetic correlations: Comparing the ‘animal model’ with parent4 offspring regression using data from a natural population. Plos One.

[b2] Benton TG, Grant A (2000). Evolutionary fitness in ecology: comparing measures of fitness in stochastic, density-dependent environments. Evolutionary Ecology Research.

[b3] Bulmer M (1980). The Mathematical Theory of Quantitative Genetics.

[b4] Caswell H (2001). Matrix Populaton Models.

[b5] Caswell H (2009). Stage, age and individual stochasticity in demography. Oikos.

[b6] Charlesworth B (1994). Evolution in Age Structured Populations.

[b7] Childs DZ, Rees M, Rose KE, Grubb PJ, Ellner SP (2003). Evolution of complex flowering strategies: an age- and size- structured integral projection model. Proceedings of the Royal Society B.

[b8] Childs DZ, Rees M, Rose KE, Grubb PJ, Ellner SP (2004). Evolution of size-dependent flowering in a variable environment: construction and analysis of a stochastic integral projection model. Proceedings of the Royal Society B.

[b9] Clutton-Brock T, Pemberton J (2004). Soay Sheep: Dynamics and Selection in an Island Population.

[b10] Coltman D, Festa-Bianchet M, Jorgenson J, Strobeck C (2002). Age-dependent sexual selection in Bighorn rams. Proceedings of the Royal Society B.

[b11] Coulson T, Tuljapurkar S (2008). The dynamics of a quantitative trait in an age-structured population living in a variable environment. American Naturalist.

[b12] Coulson T, Albon S, Pilkington J, Clutton-Brock T (1999). Small-scale spatial dynamics in a fluctuating ungulate population. Journal of Animal Ecology.

[b13] Coulson T, Catchpole EA, Albon SD, Morgan BJT, Pemberton JM, Clutton-Brock TH, Crawley MJ, Grenfell BT (2001). Age, sex, density, winter weather, and population crashes in Soay sheep. Science.

[b14] Coulson T, Benton T, Lundberg P, Dall SRX, Kendall BE (2006). Putting evolutionary biology back in the ecological theatre: a demographic framework mapping genes to communities. Evolutionary Ecology Research.

[b15] Coulson T, Ezard THG, Pelletier F, Tavecchia G, Stenseth NC, Childs DZ, Pilkington JG, Pemberton JM, Kruuk LEB, Clutton-Brock TH, Crawley MJ (2008). Estimating the functional form for the density dependence from life history data. Ecology.

[b16] Easterling M, Ellner S, Dixon P (2000). Size-specific sensitivity: applying a new structured population model. Ecology.

[b17] Ellner S, Rees M (2006). Integral projection models for species with complex life cycles. American Naturalist.

[b18] Falconer D (1960). Introduction to Quantitative Genetics.

[b19] Fortin D, Beyer H, Boyce M, Smith D, Duchesne T, Mao J (2005). Wolves influence elk movements: behavior shapes a trophic cascade in Yellowstone National Park. Ecology.

[b20] Goodnight CJ (1988). Epistasis and the effect of founder events on the additive genetic variance. Evolution.

[b21] Hairston N, Ellner S, Geber M, Yoshida T, Fox J (2005). Rapid evolution and the convergence of ecological and evolutionary time. Ecology Letters.

[b22] Jacquard A (1983). Heritability – one word, three concepts. Biometrics.

[b23] Jones OR, Clutton-Brock T, Coulson T, Godfray HCJ (2008). A web resource for the UK's long-term individual-based time-series (LITS) data. Journal of Animal Ecology.

[b24] Kruuk LEB (2004). Estimating genetic parameters in natural populations using the ‘animal model’. Philosophical Transactions of the Royal Society of London B.

[b25] Lande R, Arnold S (1983). The measurement of selection on correlated characters. Evolution.

[b26] Lebreton J-D (1996). Demographic models for subdivided populations: the renewal equation approach. Theoretical Population Biology.

[b27] Lefkovitch L (1965). The study of population growth in organisms grouped by stages. Biometrika.

[b28] Metcalf CJE, Pavard S (2007). Why evolutionary biologists should be demographers. Trends in Ecology and Evolution.

[b29] Metcalf CJE, Rose KE, Childs DZ, Sheppard AW, Grubb PJ, Rees M (2008). Evolution of flowering decisions in a stochastic, density-dependent environment. Proceedings of the National Academy of Sciences.

[b30] Ozgul A, Tuljapurkar S, Benton T, Pemberton J, Clutton-Brock T, Coulson T (2009). The dynamics of phenotypic change and the shrinking sheep of St. Kilda. Science.

[b31] Parker GA, Begon M (1986). Optimal egg size and clutch size – effects of environment and maternal phenotype. American Naturalist.

[b32] Price GR (1970). Selection and covariance. Nature.

[b33] Prout T (1971). The relation between fitness components and population prediction in *Drosophila*. II: Population prediction. Genetics.

[b34] Prout T, McChesney F (1985). Competition among immatures affects their adult fertility: population dynamics. American Naturalist.

[b35] R Development Core Team (2009). R: A Language and Environment for Statistical Computing.

[b36] Rees M, Ellner SP (2009). Integral projection models for populations in temporally varying environments. Ecological Monographs.

[b37] Roach DA, Wulff RD (1987). Maternal effects in plants. Annual Review of Ecology and Systematics.

[b38] Schuetz EG, Umbenhauer DR, Yasuda K, Brimer C, Nguyen L, Relling MV, Schuetz JD, Schinkel AH (2000). Altered expression of hepatic cytochromes p-450 in mice deficient in one or more MDR1 genes. Molecular Pharmacology.

[b39] Tuljapurkar S (1990). Population Dynamics in Variable Environments.

[b40] Tuljapurkar S, Haridas CV (2006). Temporal autocorrelation and stochastic population growth. Ecology Letters.

[b41] Tuljapurkar S, Horvitz CC, Pascarella JB (2003). The many growth rates and elasticities of populations in random environments. American Naturalist.

[b42] Turelli M, Barton NH (1994). Genetic and statistical analyses of strong selection on polygenic traits – what, me normal. Genetics.

[b43] Willis JH, Coyne JA, Kirkpatrick M (1991). Can one predict the evolution of quantitative characters without genetics. Evolution.

[b44] Wolf JB, Brodie E, Cheverud J, Moore A, Wade MJ (1998). Evolutionary consequences of indirect genetic effects. TREE.

