# Supplementary material for: Using evolutionary demography to link life history theory, quantitative genetics and population ecology
Source: J Anim Ecol. 2010 Nov;79(6):1226–40. doi: 10.1111/j.1365-2656.2010.01734.x (PMC3017750; doi:10.1111/j.1365-2656.2010.01734.x)
Supplement: Supplementary file 1 [file jane0079-1226-SD1.pdf]

Using Evolutionary Demography to Link Life History Theory, Quantitative Genetics and Population Ecology: Supplementary Information

Tim Coulson

Department of Life Sciences, Imperial College London, Silwood Park, SL5 7PY, UK

t.coulson@imperial.ac.uk

Shripad Tuljapurkar

Department of Biology, Stanford University, Stanford, CA 94305-5020, USA

tulja@stanford.edu

Dylan Z. Childs

Department of Animal and Plant Sciences, The University of Sheffield, Western Bank, Sheffield, S10 2TN, UK

d.childs@sheffield.ac.uk

Received ____________________; accepted ____________________

Manuscript was prepared with AASTeX

ADDITIONAL INTERPRETATION OF RESULTS DISPLAYED IN FIGURE 3

Increasing the intercept of the character-survival functions does not influence the covariance between a character and survival but increases mean survival rates. The magnitude of the consequences of doing this differs across age-classes. Increasing survival rates by increasing these intercepts increased population growth and generation length and decreased heritability and viability selection. Heritability decreased because more individuals survived to older ages where the association between mean offspring body mass and parental body mass was weaker than in younger age classes (Figure 1). The reason increasing mean survival while keeping the covariance between a character and survival constant decreases viability selection can be understood by writing the selection differential as the ratio of the covariance to mean survival by definition increasing the denominator while holding the numerator constant will decrease the ratio. Effects were strongest in age-classes where viability selection was greatest (Figure 1).

Increasing the slope of the character-survival functions increases both the covariance between a character and survival, and mean survival. The increase in survival rates acted to increase population growth and generation time and decrease heritability via the same processes as increasing the intercepts. In lambs and senescent individuals increasing the slope had a proportionally equivalent effect on the covariance between body mass and survival as it did on mean survival. There was consequently little effect on the viability selection differential. In yearlings and adults perturbing the slope had a greater impact on mean survival than on the covariance and this acted to decrease the strength of viability selection.

Increasing the intercept of the character-fertility functions had no influence on the covariance between the character and fertility but increased mean fertility. This acted to increase population growth simply because more individuals are being born. Whether perturbing fertility slopes increased or decreased generation time, heritability and the strength of viability selection were age-class specific. Heritability increased when fertility rates were increased in ages where there

was a strong positive relationship between mean offspring and parental body mass, and decreased it otherwise. Increasing fertility rates increased the number of offspring being born into each character class, which had knock on effects throughout life cycle influencing the total strength of viability selection. Increasing fertility rates in lambs had a large positive effect on the strength of viability selection estimated across the life cycle. Effects in other age-classes were small.

The mean and variance of body mass distributions were not strongly influenced by perturbing any parameters in the character-survival or character-fertility functions. Again, this is primarily because of knock on effects through the life cycle meant there was little change to the stable age-character distributions.

Increasing intercepts and slopes in the mean growth rate functions had effects on all quantities. Increasing growth rates obviously leads to larger individuals, which leads to an increase in the mean body mass across the population. It also leads to greater between age-class differences in mean body size that increase the variance in body mass. In all age-class there is a survival and fertility advantage of being large. Increasing growth rates consequently also increases population growth by increasing mean survival and fertility rates, which in turn increases generation length. Increasing mean growth rates also influenced the overall strength of viability selection by altering both the covariance between body mass and survival and mean survival rates. Different patterns were observed in different age-classes depending upon whether the covariance between body mass and survival or mean survival was altered the most. Increasing survival rates, by increasing growth rates, led to an increased proportion of the population in older age-classes, where the association between parental body mass and mean offspring body mass was relatively weak (Figure 1). This change in structure tended to decrease the heritability estimate.

Increasing the intercepts of the mean difference between parental and offspring character values means average offspring size is increased. These larger offspring have higher survival rates, which acts to increase population growth. These increases in survival rates impact viability

selection and trait heritability, but have little impact on any other quantities. As with survival, the consequences of altering mean offspring body mass on the heritability differs among age-classes. Altering the slope of the relationship between parental and offspring body mass increases the heritability in lambs and yearlings; there are little effects in older age-classes. The reason for this is the increase in lamb survival rates that result from an increase in mean offspring act to reduce heritability estimates for the reasons described above, while the increase in the slope tends to make offspring more similar to their parents. These counteracting effects nearly exactly cancel each other out.

Increasing the intercept of the variance associations used to construct the transition kernels increases rates of stasis individuals are unlikely to transition between very different character classes. Increasing the slope of these variance associations decreases stasis among classes with large character values. Perturbing parameters in the variance functions does not influence mean growth rates between year t and $t+1$, or in the mean difference between parental body mass and offspring body mass. Altering parameters essentially had no influence on any quantity with the exception of the strength of viability selection. Increasing the rate of mixing by perturbing both intercepts and slopes in these variance associations increased the covariance between the character and survival acting to increase the overall strength of viability selection. The lack of effect on other quantities is interesting. The degree of mixing is captured in statistical models by fitting individual as a random effect into mixed models. Our results suggest that varying the strength of persistent individual differences has tiny effects on the estimation of most quantities that interest population biologists.

Our results demonstrate how subtle effects of altering a parameter in a function can be. In many cases, altering a parameter in one function can influence survival and fertility rates and the number of individuals making particular transitions within the life cycle. It would be impossible to identify such subtleties working with only one component of fitness, or with a measure of

fitness like lifetime reproductive success.

Parameters used in the construction of the IPM

Survival, recruitment and litter size were generalised linear models with a binomial error structure. Parameters are in lists of four. The first parameter in each list is for lambs, the second for yearlings, the third for prime-aged adults, the fourth for senescent individuals.

SURVIVAL PARAMETERS

-3.5652572, -2.298034, 0.1900181, -5.6054482 – SURVIVAL INTERCEPTS

0.2801004, 0.2552713, 0.1030863, 0.2841259 – SURVIVAL SLOPES

RECRUITMENT PARAMETERS

-4.2702348,-5.6249537,-0.473731707,-3.48380923 – RECRUITMENT INTERCEPTS

0.1571317,0.2404116,0.004208452,0.09215088 – RECRUITMENT SLOPES

TWIN RATE PARAMETERS

-1000,-1000,-6.333452,-10.871048 – LITTER SIZE INTERCEPTS

0,0,0,0.2860888 – LITTER SIZE SLOPES

The transition kernels were parameterised using linear models. Once again, parameters provided in lists of four.

PARAMETERS FOR ONTOGENETIC DEVELOPMENT KERNELS

7.6561033,8.2248039,6.1479333,4.4693660 – MEAN GROWTH RATE INTERCEPTS

0.7236385,0.7108617,0.7504923,0.7929918 – MEAN GROWTH RATE SLOPES

2.39297,1.1556, -1.1010, 10.0938 – VARIANCE IN GROWTH RATE INTERCEPTS

0.04811, 0.1063, 0.1617, -0.2845 – VARIANCE IN GROWTH RATE SLOPES

OFFSPRING-MOTHER DIFFERENCE KERNELS

c(1.0891092,6.4214297,9.0065115,13.53349697 – MEAN OFFSPRING-MOTHER DIFFERENCE INTERCEPTS

c(0.5015036,0.2941641,0.1651943,-0.04821571 – MEAN OFFSPRING-MOTHER DIFFERENCE SLOPES

c(5.36041, -1.1985, -1.3743, 2.28464 – VARIANCE IN OFFSPRING-MOTHER DIFFERENCE SLOPES

c(-0.06596,0.2729, 0.2863, 0.08917 – VARIANCE IN OFFSPRING-MOTHER DIFFERENCE SLOPES

See text for details on how these parameters are used to construct the IPM and the matrix approximation of it.

Figure S1. Structure of the $\mathbf{S}(t)$, $\mathbf{R}(t)$, $\mathbf{G}(t)$, $\mathbf{D}(t)$, $\mathbf{\Psi}$ and $\mathbf{\Gamma}$ matrices. Grey cells represent elements that can be non-zero while elements in white are always 0. The square highlighted with the dotted lines represent matrix elements associated with one age-class (age 2).

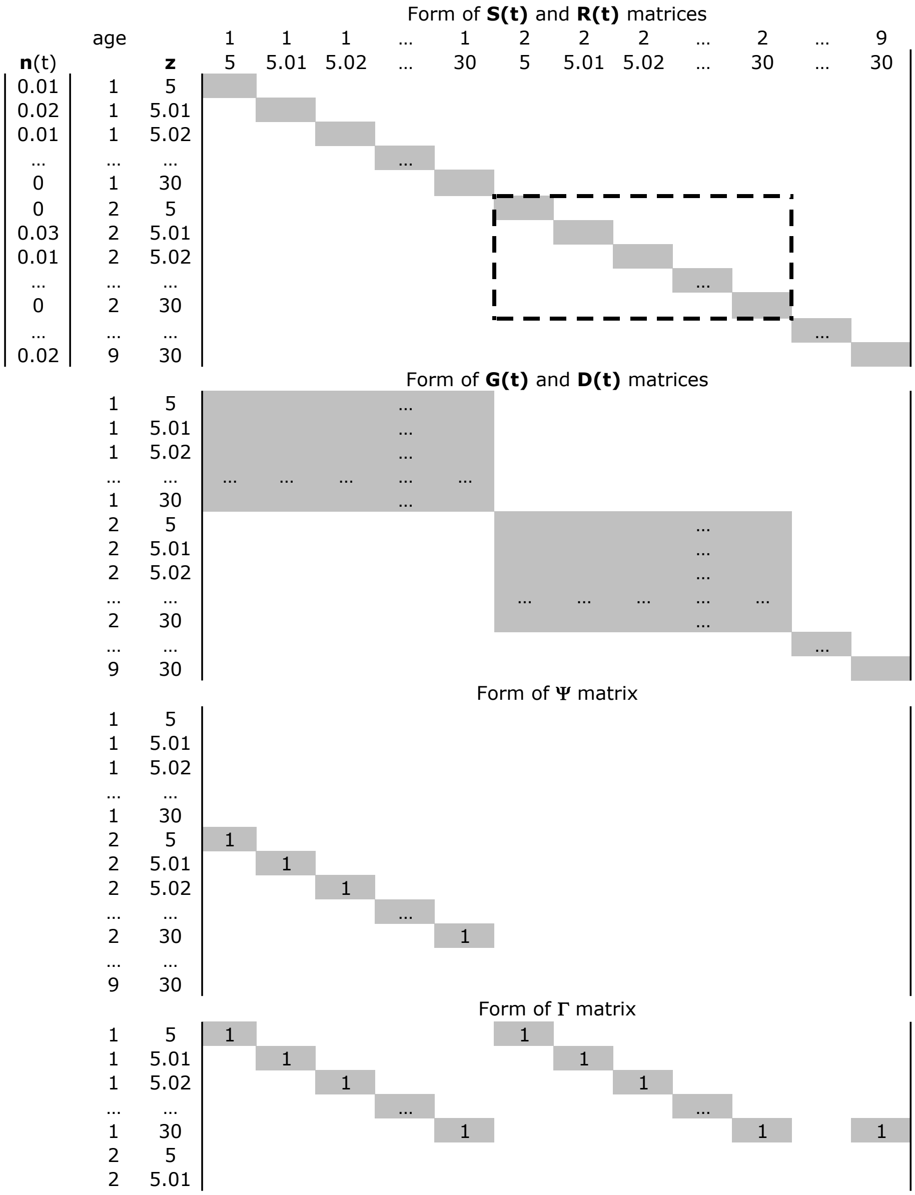
Form of S(t) and R(t) matrices
age 1 1 1 … 1 2 2 2 … 2 … 9
n(t) z 5 5.01 5.02 … 30 5 5.01 5.02 … 30 … 30
0.01 1 5
0.02 1 5.01
0.01 1 5.02
… … …
0 1 30
0 2 5
0.03 2 5.01
0.01 2 5.02
… … …
0 2 30
… … …
0.02 9 30
Form of G(t) and D(t) matrices
Form of Ψ matrix
Form of Γ matrix
