# Supplementary material for: Using evolutionary demography to link life history theory, quantitative genetics and population ecology
Source: J Anim Ecol. 2010 Nov;79(6):1226–40. doi: 10.1111/j.1365-2656.2010.01734.x (PMC3017750; doi:10.1111/j.1365-2656.2010.01734.x)
Supplement: Supplementary file 2 [file jane0079-1226-SD2.pdf]

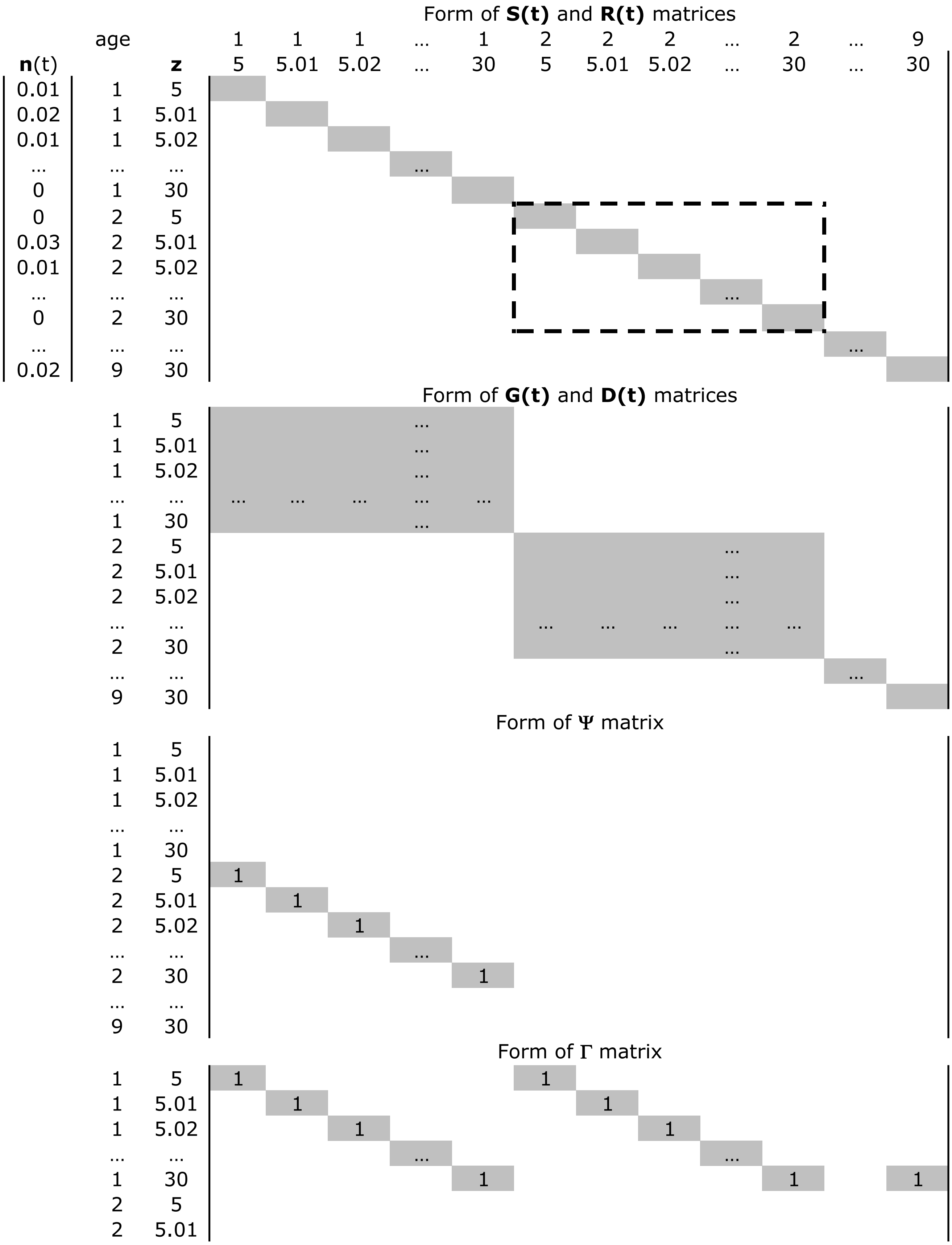

Form of S(t) and R(t) matrices
age
n(t)
z
0.01 0.02 0.01 … 0 0 0.03 0.01 … 0 … 0.02
Form of G(t) and D(t) matrices
Form of Ψ matrix
Form of Γ matrix

2	5.02
...	...
2	30
...	...
9	30
